# How physician relationships with neurotechnology companies impact clinical practice: interviews with physicians

**DOI:** 10.3389/fnins.2026.1926799

**Published:** 2026-09-03

**Authors:** Meredith V. Parsons, Judith M. Mwobobia, Erin D. Solomon, Michael Kudom-Agyemang, James M. DuBois, Tristan McIntosh

**Affiliations:** Bioethics Research Center, Division of General Internal Medicine, Washington University School of Medicine, St. Louis, MO, United States

**Keywords:** clinical decision-making, conflicts of interest (COI), medical device companies, physician-industry relationships, qualitative interviews, research ethics, technology adoption in healthcare, neurotechnology

## Abstract

**Introduction:**

Physicians and neurotechnology companies each play a critical collaborative role when integrating neurotechnology into clinical practice. Physicians serve as gatekeepers and decision-makers in adopting neurotechnology, while companies often support the development, training, and maintenance of these devices. Relationships with industry require physicians to navigate complex clinical, ethical, and technological factors that affect their clinical roles. To understand physicians’ relationships with neurotechnology companies and facilitate responsible collaborations between the two, this study investigated physicians’ perspectives on these relationships and their influence on clinical practice.

**Methods:**

Physicians (*N* = 13) using neurotechnology to treat patients participated in a semi-structured interview exploring perspectives on neurotechnology companies and how these relationships affect their decision-making and approach to patient care. Interviews were recorded, transcribed, and coded using an inductive thematic approach.

**Results:**

Physicians found their relationships with neurotechnology companies both beneficial and essential for device implementation in patient care. However, physicians acknowledged tensions that can arise when financial motivations of companies influence patient care. Company-sponsored research, events, marketing materials, and aggressive sales practices were viewed as potential sources of undue influence. But physicians also noted that reliable customer support, strong evidence of device safety and efficacy, streamlined implementation workflows, and devices that fit patients’ needs were all positive factors. Physicians expressed frustration about lack of transparency from companies regarding device troubleshooting and data sharing, high turnover in customer support or company leadership roles, and discontinuation of essential products or services. Additionally, physicians noted serious ramifications to patient care when a company discontinues a device or goes out of business without clear resolutions. Finally, physicians shared strategies for promoting objective clinical decision-making when working with companies, such as prioritizing patient wellbeing, using independent data sources, and conferring with colleagues to inform decisions.

**Conclusion:**

Relationships between physicians and neurotechnology companies are essential for connecting devices to patient treatment. Physicians, companies, and hospitals all play a role in balancing financial motivations that intersect with patient care priorities. Findings from this study underscore the importance of ensuring that neurotechnological advancements prioritize patient well-being amid the complex partnerships between companies and physicians.

## 1 Introduction

Neurotechnology research efforts to treat neurological and psychiatric conditions have skyrocketed over the last decade, and the neurotechnology market is projected to substantially increase in coming years (Elias et al., 2021; Neurotechnology Market Report by Product Type, 2023). Neurostimulation techniques such as deep brain stimulation (DBS), transcranial magnetic stimulation (TMS), and vagus nerve stimulation (VNS) have been increasingly adopted in clinical practice with great benefit to patients with severe or treatment-resistant conditions (Chail et al., 2018; Edwards et al., 2017). While other neurotechnologies like brain-computer interfaces (BCIs) have been comparatively less common in patient care than in research, enthusiasm for such research and development suggests that BCIs too may see increased clinical uptake in coming years (Sah et al., 2024; Schiff et al., 2024). The rapid growth of neurotechnology research and its potential clinical benefit highlights the need to prepare professional stakeholders in healthcare to effectively integrate these devices into clinical settings.

Physicians and neurotechnology companies hold essential roles when implementing neurotechnology in clinical practice. Physicians serve as decision-makers, selecting which devices to include in clinical offerings based on clinical evidence. They also serve as gatekeepers, advocating for the best interests of their patients. Meanwhile, neurotechnology companies facilitate device manufacturing, programming, and maintenance. Through research funding, consulting, and ongoing feedback, physicians and device companies frequently collaborate to continually improve medical devices to benefit patients and clinical operations (Chatterji et al., 2008). Further, neurotechnologies are often technically complex, requiring substantial training for physicians provided by companies in order to use these devices effectively (Chatterji et al., 2008).

These circumstances require physicians and neurotechnology companies to collaborate at various stages throughout the translational process, including research design, disseminating findings, purchasing, implementation, technical support, and ongoing evaluation. Thus, the scope and inherent necessity of relationships between doctors and neurotechnology companies have fundamental differences compared to relationships with pharmaceutical companies (Illes et al., 2020). These crucial relationships require physicians to manage intricate clinical, ethical, and technological factors that impact clinical decision-making. For example, relationships with neurotechnology companies introduce the risk of compromising a physician’s objectivity when making clinical decisions (DuBois, 2017). This includes financial transactions from companies to physicians that create conflicts of interest, as well as unconscious biases arising from the heightened degree of contact between companies and physicians required in neurotechnology implementation (Chatterji et al., 2008; Illes et al., 2020). Regardless of whether physician-industry relationships include a financial component, frequent interactions with device representatives can influence clinical decisions to favor certain procedures or devices, which may be of greater financial benefit to the company (McIntosh and DuBois, 2020).

Physicians are tasked with navigating diverging priorities between industry and medicine, balancing their obligations to provide patient care with industry partners’ needs for profit and efficiency. Further, productive and responsible relationships with neurotechnology companies require transparent communication regarding the evidence behind a device and the impact of device implementation on clinical operations and patient care (Datta Burton et al., 2021; Parsons et al., 2025). Failure to responsibly navigate differing priorities or transparently share information between parties can undermine a potentially valuable relationship between physicians and neurotechnology companies, preventing beneficial devices from reaching patients who need them most.

Relatively little is known about how physicians perceive and navigate their relationships with neurotechnology companies (McIntosh and DuBois, 2020). Given the rapid expansion of neurotechnologies in clinical care, understanding physicians’ experiences is essential for identifying challenges in neurotechnology implementation and solutions that can facilitate productive and responsible physician-industry relationships (Elias et al., 2021; Neurotechnology Market Report by Product Type, 2023). We conducted semi-structured interviews with physicians to understand their experiences and perspectives on relationships with neurotechnology companies and the influence these relationships have on clinical practice.

## 2 Materials and methods

### 2.1 Participants and recruitment

Multiple recruitment channels were used to ensure a diverse and relevant sample of physicians. The study team leveraged purposive and snowball sampling, as well as referrals from our professional networks and project advisory board to disseminate recruitment materials to potentially eligible physicians (Nyimbili and Nyimbili, 2024). Physicians’ contact information was also collected from publicly available sources (e.g., ClinicalTrials.gov, NIH RePORTER, OpenPayments). Potential participants with available email addresses received up to four recruitment emails containing a link to a screening survey administered via Qualtrics.

The screening survey included informed consent information, followed by questions to determine participant eligibility and collect basic demographic information. Survey responses were reviewed by the study team to determine eligibility and select individuals for interviews. Physicians were eligible to participate if they: (1) practiced medicine in the United States, (2) treated patients using neurotechnological interventions (e.g., deep brain stimulation, transcranial magnetic stimulation, brain-computer interfaces, spinal cord stimulation), and (3) had current professional relationships with neurotechnology industry partners (e.g., consulting, research, speaking engagements).

The study team reviewed survey responses of the 17 physicians who responded; all were eligible to participate. These 17 physicians received up to 4 emails inviting them to schedule an interview. Fifteen physicians scheduled an interview appointment, however, two cancelled their appointment due to scheduling challenges. The resulting sample included 13 physicians, a size generally sufficient to reach inductive thematic saturation (Guest et al., 2006, 2020; Moore et al., 2026; Young and Casey, 2019).

### 2.2 Data collection

Semi-structured interviews were conducted over Zoom. Each recorded interview lasted 1 h and was professionally transcribed. Interviewers included three team members with prior experience conducting qualitative research and received project-specific training from the Principal Investigator (PI) to ensure consistency and subject matter familiarity. Participants received a $60 gift card as compensation for their time.

Interview guides were informed by the literature on physician-industry relationships and developed by the study team in collaboration with the project advisory board, which consisted of physicians, patient advocates, neuroethicists, and other key neurotechnology stakeholders (DuBois, 2017; Klein et al., 2015; McIntosh et al., 2022). Questions in the interview guide covered several domains, including physicians’ professional experience with neurotechnology and industry relationships, ethical and practical challenges in industry collaboration, and perspectives on policy and patient care. Interview guides and transcripts from this study are available through the Qualitative Data Repository (QDR) at Syracuse University (McIntosh, 2022). Throughout data collection, the study team met weekly to review transcripts and assess inductive thematic saturation, evaluating whether new themes were still emerging in interviews (Guest et al., 2006, 2020; Moore et al., 2026). This study was approved by the Washington University in St. Louis Human Research Protection Office (IRB ID #202205141).

### 2.3 Data analysis

The study team employed an inductive thematic approach to analysis (Tolley et al., 2016; Vaismoradi et al., 2013). A codebook was iteratively developed by first reviewing a random subset of five transcripts to identify initial themes. The codebook was then tested on two additional transcripts and iteratively refined through team discussion and consensus. The final codebook reflected recurring themes regarding physicians’ professional experience with neurotechnology and industry relationships, ethical and practical challenges in industry collaboration, and perspectives on policy and patient care. The codebook also contained broader “other” codes to ensure that unique or uncommon themes were captured in addition to common thematic codes. Two coders applied the finalized codebook to transcripts in Dedoose software for qualitative data analysis. To ensure consistency, interrater reliability for both coders was assessed using the Dedoose Testing Center against a gold-standard coder after five and again after nine transcripts were coded. Discrepancies in coding were resolved through team discussion until a kappa above 0.80 was achieved. Once code applications were finalized, team members reviewed the excerpts for each code to generate code summaries identifying overarching themes and subthemes relevant to the study aims.

## 3 Results

In what follows, we present descriptive data to characterize our sample of physicians, followed by key themes and illustrative quotes regarding the nature of physician-industry relationships from the perspective of physicians. We examine physician-industry interactions at different stages of the translational pathway and their impact on clinical operations and decision-making. We conclude with strategies and training needs reported by physicians.

### 3.1 Sample characteristics

In addition to treating patients using neurotechnology, the vast majority of physicians (*n* = 12, 92%) were also engaged in research involving neurotechnology industry partners. These research collaborations primarily involved physicians providing consultation to industry partners (*n* = 5, 38%), companies providing lab equipment or other resources (*n* = 3, 23%), and physicians conducting non-industry funded research that involved an industry partner, such as serving as a site PI or collecting data for clinical trials (*n* = 3, 23%). A few physicians had received research funding from industry partners (*n* = 2, 15%), and two had founded a neurotechnology startup company.

Most physicians (*n* = 9, 69%) had experience using implanted brain stimulation devices (e.g., DBS), followed by implanted brain-computer interfaces (*n* = 6, 46%), and non-invasive brain-computer interfaces (*n* = 6, 46%). Others had used non-invasive brain stimulation devices such as transcranial magnetic stimulation (*n* = 5, 38%), neural prosthetics (*n* = 1, 8%), or cochlear implants (*n* = 1, 8%). Physicians reported using these devices to treat conditions such as epilepsy (*n* = 6, 46%), depression (*n* = 6, 46%), and Parkinson’s disease (*n* = 4, 31%). Full demographic information is presented in Table 1.

**TABLE 1:** Demographic characteristics of physician participants (*N* = 13).

Response option	Count	Percent
Clinical involvement with neurotechnology industry*		
Industry representative provided training on a neurotechnology	11	85%
Industry representative recommended a neurotechnology	7	54%
Industry representative provided a meal or other social event	9	69%
Physician invited to give a talk to industry members about neurotechnology	5	38%
Other (i.e., worked with industry representative in context of clinical practice)	1	8%
Types of neurotechnology companies*		
Startups	4	31%
Venture capital	2	15%
Large, well-established companies	13	100%
Nonprofit companies	1	8%
Other	1	8%
Clinical work setting*		
Academic medical center	11	85%
Device manufacturer	1	8%
Veteran’s Affairs	1	8%
Other outpatient or private practice	3	23%
Types of neurotechnology used in clinical work*		
Non-invasive brain stimulation device	5	38%
Surgically implanted, invasive brain stimulation device	9	69%
Non-invasive brain computer interface	6	46%
Surgically implanted, invasive brain computer interface	6	46%
Cochlear implant	1	8%
Neural prosthetic	1	8%
Conditions treated with neurotechnology*		
Alzheimer’s disease or dementia	1	8%
Chronic pain	2	15%
Essential tremor	2	15%
Epilepsy	6	46%
Hearing loss	1	8%
Parkinson’s disease	4	31%
Psychiatric disorders (e.g., depression, anxiety, OCD)	6	46%
Stroke	1	8%
Traumatic brain injury or concussion	2	15%
Years of clinical experience using neurotechnology to treat patients		
10 years or less	7	54%
11–20 years	4	31%
More than 20 years	2	15%
Gender identity		
Male	8	62%
Female	4	31%
Prefer not to answer	1	8%
Age		
30–39 years	3	23%
40–49 years	5	38%
50–59 years	5	38%
Race*		
Asian	3	23%
Black or African American	1	8%
White	9	69%
Prefer not to answer	1	8%
Ethnicity		
Hispanic or Latino	0	0%
Not Hispanic or Latino	12	92%
Prefer not to answer	1	8%

*Indicates a survey question that allowed multiple response options.

### 3.2 Essential collaborations with differing priorities

Physicians shared their perspectives on the necessity of industry relationships and noted differing priorities between companies and clinical practice.

#### 3.2.1 The beneficial role of industry partnerships in innovation and treatment development

All physicians (*n* = 13, 100%) noted at least one beneficial aspect of their relationships with neurotechnology companies in the context of research or clinical care. Relationships with device companies were seen as essential for translating scientific discoveries into treatments that benefit patients. Working with companies provided physicians with access to the newest technologies, or the potential to adapt existing ones, enhancing their clinical offerings to patients.

*“Particularly when we want to innovate and develop new treatment strategies, we have to work with these partners to be able to use the newest technology or even potentially adapt the technology that they already have.” (Physician 12)*

*“I mean, the biggest benefit is that they [device companies] can do the research faster. They can innovate faster than we can with the amount of funding that research has in universities…We need industry partners in order to translate the work that’s done on our campuses into something that can get into patients’ hands in a safe way.” (Physician 02)*

#### 3.2.2 Differing priorities between industry and medicine

While physicians recognized that device companies play an essential role in clinical contexts, the majority of physicians (*n* = 9, 69%) highlighted challenges with industry collaborations–primarily revolving around differing goals and priorities between clinical and commercial environments. Physicians shared that their top priorities were patient care, safety, and data privacy. However, they observed that industry partners were primarily driven by financial incentives surrounding the commercialization of neurotechnology devices and sustainability of the company. Physicians observed these financial motivations causing company representatives to pursue short-term positive outcomes over long-term benefit, such as prioritizing new device sales over maximizing the number of treatments per patient.

*“In the world I work in, the only thing that industry cares about is the bottom line, so 95 percent of their interest is financial reimbursement…the people that are driving all the innovation are essentially have to be at the medical device [companies], and whether or not they choose to do the right thing has nothing to do with academia. It has nothing to do with the FDA…I largely believe that the people who are there at each of the device companies are generally good people, but as a whole they start making terrible decisions because it’s mostly driving by short-term outcomes.” (Physician 03)*

*“…I think the problem we had with [Company] is that sometimes the salespeople for [Company] were incentivized to sell more devices. They’re really not incentivized to maximize the number of treatments…Sometimes they would be partnered with us, but they would also undermine our efforts to treat a lot of patients and have a lot of patients on our [TMS] machines. Or they’d encourage other people to compete with us, even while we supposedly had a good relationship with them.” (Physician 04)*

#### 3.2.3 Research and patient care compromised by physician dependence on device companies

In terms of impact, five (35%) physicians specifically noted how differing priorities between device companies and physicians can strain relationships that physicians depend on for both professional collaboration and patient care. First, physicians acknowledged that they are disproportionately reliant on companies that control access to essential hardware and software required for both clinical and research endeavors. Physicians also noted that profit motivations of companies influence the types of research and development projects that companies fund. For example, companies favor projects with significant commercial prospects over potentially valuable scientific inquiries focusing on smaller patient populations. Physicians speculated that these limitations affect the number and scope of neurotechnology devices that ultimately become available for patient care, especially for rare diseases. Finally, after a device is developed and brought to market, physicians discussed how they rely on industry representatives for critical support in troubleshooting device issues and adjusting treatment parameters. Clinical operations suffered when insufficient training, documentation, or technical support was provided by the company.

*“It’s a real pain to work with these companies primarily because there is an inherent power dynamic that exists. It’s funny because a lot of times, as a clinician, you supposedly have a lot of power when you’re talking to these device companies because they want your business, right? They don’t wanna make enemies out of people. While that’s true, it really is a miniscule amount of power because the majority of power rests with these companies…It’s not a great sort of environment, I think. The medical device company, unlike a lot of the pharmaceutical companies, they essentially maintain all of the power around the devices. We need the devices in order to be able to do any kind of clinical work or research, and they use that power in a lot of ways that we cannot control. Whereas, if a drug is FDA approved, and you wanna do a study or you wanna develop a whole area, it’s possible to do that without 100 percent cooperation with companies because they become generic over time. Whereas devices, they do not in many ways, and so you are beholden to the ever-changing feelings of medical device companies in a very real way. It is extraordinarily painful to be in this field.” (Physician 03)*

### 3.3 Influence of industry marketing and business objectives on clinical outcomes and decision-making

Physicians noted several ways in which the marketing or business priorities of companies influence clinical outcomes and decision-making.

#### 3.3.1 Physicians vary in their perception of the role bias plays in clinical decision-making

Physicians (*n* = 7, 54%) shared a range of perspectives regarding the presence of biases emerging from industry-physician relationships and their impact on clinical decision-making. Physicians who acknowledged bias emphasized needing awareness of personal and systemic biases, promoting continued professional education and transparency with patients to mitigate these biases. They noted that in general, the mere presence of device representatives can inherently influence clinical decisions such as prescribing patterns or how often a particular treatment is recommended to patients. This included having device representatives being physically present in clinics and operating rooms, meetings, or conferences, as well as the distribution of company-branded items such as pens, tote bags, and other giveaways. These physicians recognized that biases are an inherent part of being human and strived to counteract them through introspection and open communication.

In contrast, other physicians did not see biases associated with industry influence as a significant problem in their decision-making processes, viewing their choices as primarily driven by objective assessments of patient needs and the clinical evidence available. These physicians relied on personal heuristics and institutional checks, such as case conferences, to maintain objectivity.

*“I think that in general, medicine is not very good at appreciating the biases everywhere. I’m smart, so I’m unbiased. That would be nice, wouldn’t it? I feel like that attitude is prevalent that most physicians don’t think that having drug reps provide samples biases them and when obviously it does. If you have samples of something, you’re more likely to say, oh, why don’t you try this? Then that leads to more sales of that drug versus its competitors. If you have heard more about a drug, because it’s talked about more in your presence, then you’re more likely to prescribe it.” (Physician 13)*

*“Companies make gizmos, and my job is to choose the right gizmo for the right patient. I don’t see it any more complicated than that, right? I have a panoply of devices that these devices have available for me and I can choose. I go to the store, and I choose which device I think is best for the patient. I implant that particular device, right? I recognize that at the outset, that there there’s implicit bias with regards to one particular device over the other. Frankly speaking, in terms of my decision making, I have developed my own algorithms and my own heuristics for patient care that are company agnostic with regards to device implantation.” (Physician 10)*

#### 3.3.2 Industry marketing strategies or engagement at conferences can influence clinical decision-making

Physicians (*n* = 6, 46%) observed that industry-sponsored conferences, events, and materials tend to be more enticing than their nonprofit counterparts, with features like charismatic emcees or free, branded, giveaways. Further, these physicians noted that the type of person who gets hired as a medical device representative are those with charming, magnetic, and persistent personalities. Physicians felt that the inherent sales and marketing objectives of companies motivated the flashy presentation of information to physicians learning about novel neurotechnologies that may benefit their patients. Physicians acknowledged that these marketing tactics have the potential to influence clinical decision-making when style of presentation is prioritized over quality and transparency of empirical information shared about a device.

*“I think the biggest thing is that they, like doctors in general, are not well trained to really evaluate primary data and evidence. That’s what I do in research. I feel like because of that, I’m able to read the primary literature. Regular private practice doctors just from medical training and residency…if the only thing that is easy to read is their [the company’s] reading materials, which are obviously biased and say they’re biased. If you don’t have any counter reading materials. It’s hard to get good data for yourself when you’re busy and don’t know how to do it well. I have a whole lot of empathy for the private practitioners who are just trying to–their hearts in the right place, but they don’t have the right knowledge base. All they can do is go with whatever is put in front of them. The glossy, pretty stuff that’s easy to understand, that’s put in front of them is from the from industry.” (Physician 13)*

*“I agree that funding speakers, funding projects, funding any kind of funding or support is like to bias physicians, but it’s tricky because funding and support from non-private sources is not readily available. You’re in this tricky position, if you do want to do research in these fields…either you do it all yourself or you ask them to at least put some of the data together for you in a clean format. Which I think is minimally biasing the results, but still you’re publishing on this product and you’re getting the word out there. At least in the papers that I’ve written I’ve talked about the positives and negatives, but I’ve still wrote about them probably more focused on the positives than the negatives.” (Physician 05)*

#### 3.3.3 Competitive business tactics in industry do not serve the needs of patients or physicians

Several physicians (*n* = 5, 38%) expressed distaste with the business tactics of some device companies. While many physicians were happy with the support provided by their industry partners, they observed that some companies employ aggressive sales techniques that prioritize selling large numbers of devices, rather than less profitable strategies that maximize treatment for the greatest number of patients. Additionally, physicians noted that device pricing set by companies can include hidden fees for essentials, such as clinical staff training, ongoing technical support, and necessary supplemental materials like software or fee-per-treatment options (e.g., with TMS). Physicians felt these costs should be included in the initial price of devices, especially since current pricing models can make it particularly difficult for uninsured and underinsured patients to afford these treatments.

*“I think some reps for companies–I’ll name names–like [Company 1], very aggressive marketing. Sometimes unethical, I think, marketing. For example, I got an email saying, “please distribute this dinner option to your fellow trainees during our national meeting.” I went to follow the link where they were collecting the information…they were just trying to fish for information. It didn’t even seem like there was a dinner…I think the tricky thing, unlike drug reps where you don’t need them, you can refer to your pharmacists and you can do your own research, when it comes to a device, technology, you’re a physician, not an engineer. You rely on representatives from the company to make sure they’re working properly. You can’t function without them. It is a codependent relationship.” (Physician 05)*

*“When the patient is in the operating room…that implant that’s going in will give us some feedback about its proximity to the nerve we’re trying to stimulate. It will help the surgeon maybe reorient that implant or just be able to run tests to make sure everything’s working, that the nerve is responding well. We have one manufacturer right now that’s charging–or wants to charge us to be able to have access to that equipment. That does not seem right. If it’s something that’s required to try and do a better job and service our patients better, these are things that the manufacturers should automatically provide.” (Physician 01)*

#### 3.3.4 Favoritism between physicians and companies can influence clinical outcomes

A few physicians (*n* = 3, 23%) noticed that companies sometimes select “favorite” clinical sites or providers, granting these sites better support or software and data access packages. As a result, other sites, especially those with less funding, encounter more challenges obtaining industry funding or technical support essential to patient care, while favored sites are more likely to use well-supported devices. In some cases, physicians reported companies incentivizing a physician’s competitors to buy and offer more devices, further complicating the landscape for patient care. By the same token, other physicians reported developing “favorite” companies due to long-term use of a device, positive past experiences with the company, or personal connections within the company. This preference can shape their decision-making and inclination toward using certain devices over others.

*“There are real limits to what industry is going to make available, depending on how well a study is aligned with their long-term promotional interests. They may have a technology that would actually be great for study and great for participation, and simply say, no, because they don’t want the risk associated with having that device going to a patient and be in that patient for very long term. They tend to only take on that risk if it’s somewhere where there’s a big money-making opportunity for them, or the person making the request is a very favorite client of theirs for their existing product and it’s a VIP who they wanna treat nicely. That’s where industry profit incentives absolutely constrain the kinds of science that can be done.” (Physician 06)*

*“If you get pulled into the only one company is good at this thing and you’re really using only one company, or you’re conflicted in other ways…Those are the places that we have to make sure that physicians are mitigating their risk as much as possible. It is easy enough to say to physicians, “Hey, don’t buy stock in these companies. When you do research with these companies, you’ve got to make sure you disclose”…What does it look like to manage any of these conflicts and for our patients to know when we’re conflicted as we help them make the choice about what device they’re going to use?” (Physician 02)*

#### 3.3.5 Information sharing between companies and physicians can have both positive and negative influence on clinical outcomes

Physicians (*n* = 5, 38%) also mentioned frequently collaborating with device representatives to exchange resources and information, which can both positively and negatively impact clinical decisions. For instance, a physician might consult with a device representative for training or to gain a better understanding of a device’s operations, potentially enhancing patient care. Conversely, device representatives may lack understanding of the clinical context, making it difficult to provide accurate and nuanced information that is suitable for an individual patient’s needs. Regardless of whether this information exchange was a positive or negative experience, physicians acknowledged that dependence on information from industry representatives had potential to influence clinical decision-making.

*“…I know like patient safety as an example where all of the treatment guidelines say that the patient should be wearing a hearing protection during treatment but I had heard that some of the trainers from one of the manufacturers when they were doing the training, they told some of the trainees, you don’t really need to do that. It’s like, oh, just ask the patient and if they don’t wanna wear earplugs, it’s no big deal but no, it is a big deal. I think, again, we have non-medical people giving advice to medical people, and giving dumb advice, giving advice that we know is wrong. That’s…one way in which in industry might sometimes influence physicians to practice poorly if they have underqualified people giving poor advice to physicians who are new that to that technology.” (Physician 04)*

*“Yes. They [device company representatives] can influence our decisions. I have another company who has relationships with me, that’s responsive neurostimulation therapy, which is placing the electrodes in the seizure focus, and then deliver the electrical shock to treat seizures. I have that close relationship with those–that company. Then they gave me a kinda recommendations. In that sense, they can influence our treatment. However, I have never felt their recommendation is deviated from the standard of therapy. That means, their recommendation is kinda helping me, but I don’t think they are altering my treatment negative way. They are simply helping me.” (Physician 11)*

### 3.4 Clinical practice and operational impact

Physicians discussed both positive and negative impacts that relationships with industry can have on clinical practice and operations.

#### 3.4.1 Benefits of medical device representative expertise and practical support

Several physicians (*n* = 5, 38%) highlighted the valuable knowledge that medical device representatives possess about their products, serving as essential resources for the latest scientific information and advancements related to their devices. This expertise was a significant benefit to physicians’ understanding of new technologies, on-label versus off-label indications, and the functionalities of devices.

Other physicians (*n* = 5, 38%) similarly noted that the expertise of device representatives translates to valuable technical support and equipment troubleshooting. Device representatives can act as an extension of the clinical or research team, resolving issues quickly and efficiently, which is often crucial for effective use and maintenance of neurotechnology.

*“In terms of the role of the rep in the company, I value their expertise, in terms of them having worked with a variety of clinicians at various kinds of experience levels. We will often ask them for their input with regards to surgical optimization and thinking through issues that I may not have thought of myself. As an example of this, with regards to RNS–responsive neurostimulation–the reps, for example, that have seen several times more cases can not only cover my operations, they cover operations from other surgeons. They’ll provide suggestions with regards to incision planning, or perhaps device management planning, or the amount of bone to remove in order to optimize placement as part of that. That’s an example in which a rep can actually provide value in part of an operation.” (Physician 10)*

*“I would say the one thing that I’ve found to be very helpful, with our field in particular, it’s been growing more rapidly than we have staffing for. They [device representatives] have been taking up more of the actual equipment troubleshooting, and if people need to order supplies, if people need tutorial videos about how to work with their equipment, it’s been the manufacturers’ team that has been really stepping up to take some of that burden so then we can do things more as a clinician would do.” (Physician 01)*

#### 3.4.2 Poor transparency between companies and physicians poses challenges for patient care and research

Nearly half of physicians (*n* = 6, 46%) noted significant transparency challenges when working with industry partners. Physicians expressed frustration that identifying and reporting technical issues or software bugs often falls on physicians. They felt that companies sometimes lack straightforward solutions or answers to questions or lack follow-up about the steps being taken to address reported concerns. For example, one physician shared that a device company had not provided sufficient data on the characteristics of patients who did not respond to device treatment despite repeated requests, leaving the physician to manage frustrated patients without clear guidance. Physicians acknowledged that sometimes companies were unable to provide information because the company had not yet collected sufficient data regarding an issue. However, some physicians were suspicious of whether companies may be withholding useful trends or information.

*“…I just wish, those statistics of one third of patients being non-responders to these neurostimulation treatments. I wish I had known the consequences of that impact on the patient before I started using these…We’d get patients coming back for years saying, “This hasn’t helped me at all. I went through all this and it hasn’t helped me at all and I’m really frustrated.” They’re tearful and they’re angry and I totally get it, and I am too… I wish we had more information on who these technologies are not gonna work for. We really don’t get that information until it’s used more widely, and again I’m not sure how transparent the companies are in sharing that information…That’s the thing that, as a provider, constantly being the one to field the complaints, understandable complaints from these patients, has made me the most reticent at continuing to advocate for these devices.” (Physician 05)*

*“I would say sometimes they’re [industry representatives] not completely able to share all the details…When we start having certain types of issues or bugs that pop up, sometimes they can’t really say too much until they start getting enough data. It’s just kind of like we’re trying to help our patient, and sometimes, certain things with the software not working the way that it’s expected to work or the apps are not working the way that they should. Sometimes, we just don’t get straightforward answers in the beginning until they’re able to come up with the information themselves, so that’s understandable. But it is frustrating.” (Physician 01)*

#### 3.4.3 Neurotechnology devices are complicated and require training to use

Some physicians (*n* = 4, 31%) mentioned the complexity of skills required by clinical staff due to the variety of proprietary device programming platforms developed by each company, some of which do not integrate with Electronic Medical Records (EMR). Thus, clinical staff are required to become fluent in multiple systems and come up with work-arounds for platform limitations (e.g., manual data entry in EMR). Such efforts demand considerable time, labor, and expense to the clinical team–particularly in cases of staff turnover or changes to the device. Some physicians noted that the style of platform can also influence clinical operations and decisions, especially if the platform makes the device easier for patients and clinical staff to use.

*“We bought a [TMS] chair, now we’re trying to actually implement it. That’s not been easy…it’s really hard to do, technically. You have to get the staff trained and then you have to find the right patients and then you have to–starting a whole new initiative is not trivial…We had hired a nurse and stuff. Then she left immediately…and we were down on staff so we couldn’t start again. Then by the time we were ready to start again and hired another nurse we had to get retrained, but they had already come out to train, and so then they were going to charge us an enormous amount of money to–it’s all these moving pieces…that’s been hard.” (Physician 13)*

*“Each of the three companies has its own platform that we’re using so learning three different platforms. I’ve got three different tablets that I’ve got to use in order to program the three different devices…Documentation is hard because none of those devices connect to our electronic medical record, which means that I have to program on the device and then translate that data to the electronic medical record. Everybody figures out their own solution for that, including manual entry, which means that there’s potential sources of error. Pulling over a PDF or taking a screenshot, which means that you have data that you can’t do anything with once it’s in your medical record. Certainly, those are places where we’ve wanted to see growth and that’s been slow in growth.” (Physician 02)*

#### 3.4.4 Device quality and support influences clinical decision-making

Several physicians (*n* = 5, 38%) noted that they evaluate the quality of the device and the support provided by the company when making decisions about whether to use a particular device. For example, if clinical field engineers are not available in a patient’s home area, a physician might recommend an alternative device to better serve the patient. This was often described as one way that industry offerings can influence clinical decisions about which neurotechnology device to prescribe.

*“I think the primary factor for what device company people choose is primarily ad hoc, but number two is based off of support…In DBS, for example, the primary factor is whether or not the neurologists were trained and feel comfortable with the software… In the pain world…for example, in spinal cord stem, the only factor is whether or not you have a company rep who is willing basically to ‘do all the work for you,’ like…there’s no actual basis for it other than having someone who’s willing to answer phone calls and talk to patients and get them ready, and in some ways that should be how it is…It definitely blurs the boundaries between the company and the clinical responsibilities a lot more than in other fields.” (Physician 03)*

*“They [device companies] differentiate themselves…In the case of deep brain stimulation, the three different companies have three different platforms that have three different pros and cons that a clinician can choose from…There’s three different designs, right? A surgeon may decide they want to use design A versus design B for reasons X, Y, and Z…With regards to spinal cord stimulation, this is a crowded area with regards to, I think, about four or five different companies…a lot of it differentiates in terms of patient relationships, right? A lot of the programming that’s done as an outpatient basis is done by company representatives. The availability and the emotional intelligence and the experience of the programmers makes a dramatic difference in terms of the decision making that a clinician could have in terms of choosing one company over the other–because effectively, for the most part, they all do very similar things.” (Physician 10)*

#### 3.4.5 Companies going out of business or experiencing high levels of turnover cause significant hurdles for physicians

Roughly half of physicians (*n* = 6, 46%) reported negative experiences with device companies having employee turnover, going out of business, or ending support for devices–all of which significantly impacted physicians’ work. Physicians noted that these types of changes can disrupt ongoing patient treatments and create inefficiencies in clinical operations. Further, when companies go out of business or end support for devices, physicians and their patients can be significantly affected, left with inoperative devices and no recourse for repair or maintenance.

*“I think that they’re [device companies] doing as well as they can with the information that they have. I think they’re doing a great job, and it just depends on who your representatives are at the time. I think their customer services have been getting changed over quite a bit, so sometimes you’re back at square one if your clinic has a different way of doing things. We have to re-educate them…I do feel like customer service sometimes gets changed over, and all of the sudden, it’s like, what happened? I’m having to start over again to explain what we need.” (Physician 01)*

*“I mean, I think for one, what is the guarantee of being able to use this device and patients be able to get access to a device. I’ve definitely seen it in the past where I think people have implanted something and then the company goes under and then patients are left with a device that doesn’t work and something that they can’t fix. I mean, it definitely comes to my mind. Do I think that this is–how much, I guess, do I believe in the specific device and or the indication for the neurotech intervention?” (Physician 12)*

### 3.5 Strategies to promote objective clinical decision-making

Physicians also shared numerous strategies they employed in attempts to maintain their objectivity in clinical decisions when partnerships with industry are involved.

#### 3.5.1 Make patient care and communication the highest priority

First, many physicians (*n* = 9, 69%) emphasized making patient care as the highest priority in their decision-making. They emphasized communicating the benefits and risks of all available treatment options to patients, selecting the most appropriate device for each patient based on clinical indications and shared decision-making.

*“First and foremost, we want to provide the optimal care we can to the patient. I think what that involves is being as transparent as possible, communicating to the patient all their different options, not focusing on one and explaining the positives and negatives of the different neurotechnologies. Fortunately, in epilepsy, it’s not like we have just one, now we have three different neurostimulation choices if they’re not a candidate for surgery. I think we are fortunate that we have more equipoise than other fields where they might only have one option or two options. I think that helps, but I’m sure we’re all biased in how we sell different things to patients.” (Physician 05)*

*”We owe the patient the fact that we’ve done our research, we know enough information about this device that we’re gonna give you and make the claim that it’s gonna treat you. It’s not up to the patient to do that research on our behalf. Yeah, I think that’s important.” (Physician 07)*

#### 3.5.2 Seek educational resources and training

Given the various avenues in which device companies could influence clinical decision-making, many physicians (*n* = 8, 62%) emphasized the necessity of education about neurotechnology and bias, starting in medical school or even undergraduate education. They stressed the importance of training on the capabilities and ethical implications of neurotechnology, in addition to recognizing and mitigating biases that arise from industry relationships. The value of mentorship and learning from experienced colleagues was also highlighted, especially for physicians who have little or no prior experience interacting with neurotechnology companies.

*“I think it’d be useful to have classes or some sort of education on neurotechnology or even in things undergrad or high school. Not a mandatory thing, but just something that people can learn more about. Any sort of tool wouldn’t just be for medical school, but maybe ultimately for different levels of education. Inclusive, like I said, high school, undergrad, graduate school, and medicine. Law.” (Physician 12)*

*“I’d say mentorship from people that have experience with it [device company relationships] is the biggest thing. It’s not super common for people to–for that many mentors, I found to have the level of experience that’s required to really help navigate…I can pick up my own trajectory, and I love mentoring students…but because I had to give up my academic position to start my company, to make it easier to deal with the COI, honestly, I just didn’t feel like dealing with those, ends up being so complex. You run into this situation where now I have extraordinary amounts of knowledge about how the reimbursement system works.” (Physician 09)*

#### 3.5.3 Implement and follow institutional policies that support objective clinical decision-making

Physicians (*n* = 6, 46%) highlighted the role of healthcare administrators and regulatory bodies, such as the Food and Drug Administration and Institutional Review Boards, in ensuring that clinical data handling adheres to ethical standards and that patient interactions contain proper disclosures in accessible language in light of physician-industry relationships. They appreciated the existing guidelines and policies that facilitate transparent communication with patients and colleagues when an industry relationship is involved, providing a structured framework to navigate potential conflicts of interest or data quality concerns.

*“These are large risk adverse companies…everybody that I partner with is a company that wants to stay in business and make money. They depend on their reputation. They just are so disincentivized to do anything to be unsafe…With the level of FDA oversight on them, I have never had any worry about the actual safety or quality of manufacture and freedom from defects about the product through the partners I work with.” (Physician 06)*

*”Outside clinical duties, the only other people who I’ve had involved are IRB officers…They have very clear guidelines they want you to follow, especially if you are storing any patient data on some a cloud-based system…Other than the IRB, I think when I was at [University 2] we had a financial office that dealt with the money part…It was all very clearly stated, and I didn’t have to worry about how the money was shared around…The other person in the administrator…it was like a publications and like, not PR or HR, but it was like a clinical office. You would submit your paper to make sure you weren’t saying anything that is not appropriate or correct, especially if there’s a funding agency involved…We had somebody who would just check it for that, like technical stuff and making sure that we weren’t presenting anything in an inappropriate light.” (Physician 08)*

#### 3.5.4 Limit industry interaction

Physicians (*n* = 7, 54%) also reported taking deliberate measures to limit their exposure and interactions with medical device companies as a means of maintaining objectivity in clinical decision-making. By implementing and following institutional policies that prohibit or discourage industry-funded meals, events, or gifts, physicians aimed to minimize potential sources of bias that can influence their choices. Additionally, many physicians reported adopting a business-only demeanor in their interactions with industry partners, ensuring that these engagements remain focused on the technical and practical aspects of device use rather than becoming personal. Physicians felt that a cautious stance toward industry partnerships helps sustain the integrity and objectivity essential for effective and responsible clinical practice.

*”We try our hardest to maintain as business-like interaction with industry partners as possible. We don’t take gifts. We don’t take pens, shirts, anything from industry. On our desks, you will not see a pen that has [Company 1] or [Company 2] or [Company 3] on it. We don’t take trips. We do very little advising because we really have looked at the data for how these relationships impact decision-making and based on that, we say we have to be as unconflicted as possible. However, it’s impossible to be completely not conflicted because we have to be in these relationships. That means that we monitor ourselves and how much we’re engaging in these relationships and try to limit which of us are engaging in those relationships.” (Physician 02)*

*“I have no issues navigating the financial incentives with regards to these various kinds of companies. As I talked about, as a concordance with the Sunshine Act, I’m very careful with regards to–even so much as a cup of coffee, I actually pay for myself. Not every clinician feels the same way. I’m cognizant of my role in these various companies in providing the best care I can to patients. I think that I’ve tried my best to distance myself from economic incentives or possibilities from the various kinds of companies that I work with on a regular basis.” (Physician 10)*

#### 3.5.5 Seek independent data and opinion sources

Physicians (*n* = 7, 54%) often sought non-industry funded (e.g., federally-funded), peer-reviewed publications to garner a comprehensive understanding of device capabilities and limitations beyond company-provided information. Physicians noted that industry-provided data tends to emphasize the “best-case scenarios” for patients. Physicians also acknowledged that reviewing independent research about a device helps them critically evaluate industry-provided information against studies funded by non-industry sources. Physicians felt that this approach helps ensure that clinical decisions are based on a robust and complete picture of the evidence, rather than solely industry-generated data.

*”We’ve had to be very stringent as we’ve done our education for our teams here in that we typically do not go by data that is in industry sponsored papers, but rather we’re really looking at independent data that validates the outcomes of clinical trials in order for us to make choices. That’s been a growth in really looking at…what are the biases of the people that are writing the paper? Who’s getting funded by industry as they do their research? Has this been replicated by somebody who is not such conflicted in those ways?” (Physician 02)*

*”It’s been very straightforward in terms of reimbursement and who the population is. I’m pretty up front about it. I’m also very comfortable reading scientific literature too. Getting my own–I wanted to know more about efficacy data and what’s actually been tested and what’s been looked at. They [the device company] couldn’t give me that. Then I look it up myself, which is probably less bias.” (Physician 13)*

#### 3.5.6 Rely on group discussion with peers and colleagues

Nearly half of physicians (*n* = 6, 46%) reported relying on colleagues and multidisciplinary case conferences as a means of supporting objectivity in clinical decision-making. These discussions often involved a wide range of healthcare professionals, such as neurosurgeons, neurologists, neuroradiologists, psychiatrists, nurses, or social workers, who collectively review patient cases in detail to determine the best treatment options based on the balance of risks and benefits. These physicians emphasized that such collaborative environments allow physicians to critically evaluate different approaches, share insights, and challenge one another’s assumptions. Physicians felt these conferences were effective in preventing decisions from becoming unduly influenced by industry representatives.

*“Every academic epilepsy center across the country does this similarly…before any surgical decision is made, we have a multidisciplinary conference, which includes all the epilepsy faculty, neuroradiology, neurosurgery, neuropsychology, often nursing or social work. We all present the patient’s case, talk about all the data in extreme detail…and we figure out, okay, this would give the best outcome, risk versus benefit…The companies are not involved in any of this as they should not be, because they don’t have the clinical judgment to make this. It’s really the patient’s decision in the end, but it is our job to make sure the patient understands what decision we’re making for them.”(Physician 08)*

*”I think that these case conferences definitely help with objectivity and providing a relatively fair assessment of use. I think that it’s been a very beneficial, but when you’re positioned to have something like a clinical case conference, then you’re definitely able to prevent–I think it’s definitely helpful in preventing influence. Now, I’m sure that a lot of people in practice don’t have that, and so they just use what’s available or easiest to use, utilize. Again, just FaceTime is going to have an effect on utilization.” (Physician 12)*

## 4 Discussion

Physician-industry relationships are essential for bringing neurotechnology devices to patient care, and for the continued development of neurotechnologies that benefit patients. Companies bear the primary responsibility in supplying physicians with device education, technical support, and the devices themselves; this means that neurotechnology-based patient treatment could not happen without collaboration between physicians and companies (Chatterji et al., 2008).

Within these essential relationships, some physicians felt they had less bargaining power than companies, as companies control device supply chains and serve as the primary information source for physicians learning about new devices or requiring technical support. Because neurotechnology is a novel and developing field, there are many devices that do not yet have equivalent or generic competitors in the market, leaving healthcare providers more reliant on the limited suppliers that exist (Haston et al., 2025; Neurotechnology Market Report by Product Type, 2023).

The asymmetry of the partnership positions device companies to influence, either intentionally or unintentionally, multiple stages of the innovation-implementation pathway, ultimately impacting clinical outcomes. The rapid pace of novel neurotechnology innovation paired with the risk of the “valley of death,” where promising ideas fail to scale beyond a certain stage of development, makes it likely that several neurotechnology companies become insolvent or discontinue a device, increasing the risk of patient abandonment (Drew, 2022; Pienta, 2010). This risk is exacerbated by proprietary components or software packages that do not transfer to devices offered by the few competitors that do exist. Loss of access to device-supported care is a major problem for patients (Drew, 2022). This problem may improve over time as new competitors for devices enter the market. Companies operating within well-established sectors of the medical device market, such as pacemakers, have been operating and well-funded long enough to establish standards for their components and compatibility (Jeffrey and Parsonnet, 1998). Additionally, these companies have enough competitors to offer diverse products and fill smaller niches of the market (Dones, 2019).

It is essential for neurotechnology companies – particularly startup companies – to have contingency plans in place to maintain patient support in the event of device discontinuation or company dissolution (Dasgupta et al., 2024; Peabody Smith et al., 2025). To prepare for the projected growth of the neurotechnology market, companies would benefit from exploring options that ensure the long-term continued support of patients using their devices. This might entail devising universal device components or code packages across companies producing similar devices. Further, patients considering neurotechnology-based treatment need to adequately understand and be informed of important factors, including risks to long-term device access and sustainability of care related to upkeep of the device, before consenting to treatment. The evolution of the neurotechnology industry will need to be monitored by future research and may warrant consideration for future regulation to secure sustainable patient access to devices and neurotechnology-involved care.

Physicians described wide variability in company sales tactics, communication, and quality of support provided in clinical operations, revealing several strategies that neurotechnology companies can employ to strengthen clinical relationships. Physicians in this study consistently valued companies with knowledgeable, well-trained device representatives providing education and technical support. Delegation systems identifying which company personnel are responsible for physician support when concerns arise can help prevent personnel overextension or diffusion of responsibility that can cause a lack of responsiveness. Neurotechnology companies could benefit from prioritizing prompt and transparent communication when answering physician questions or resolving problems and follow up with solutions; even if they may not provide direct benefit to the physician who originally surfaced the issue, it can still be beneficial to the physician in future patient care (Morschauser, 2014).

Further, ongoing physician and patient feedback to a neurotechnology company can fuel user-engaged research that continuously improves devices for more competitive market offerings (Martin and Barnett, 2012; Olubajo et al., 2022; Shah and Robinson, 2007). In the event of employee turnover, neurotechnology companies that seamlessly transition customer accounts to new roles may prevent disruptions to patient care and clinical operations, building a sense of reliability and consumer trust. It may be beneficial for neurotechnology companies to engage in periodic self-auditing of their practices and policies to identify opportunities and areas for improvement on these factors to facilitate strong and lasting relationships with physicians and hospitals.

Physicians had mixed views about how and whether the activities of industry can influence clinical decision-making, or whether this influence is always negative. As described by physicians, there is a role for positive influence on clinical decision-making. Having a strong track record of customer support and physician education, quality evidence, best fit for individual patient needs, and strong contingency planning for financial strife are all good reasons for a physician to demonstrate a degree of “brand loyalty.” By prioritizing the patient and physician experience over flashy marketing tactics, neurotechnology companies can earn consumer trust–something that is difficult to build, easy to lose, and essential to sustaining long-term market share (Mal et al., 2018).

However, there are very real negative consequences that can arise when profit motivations from companies or physicians influence patient care decisions. While physicians emphasized their priority for patient care over financial incentives, hospitals, clinics, and neurotechnology companies alike all operate within a capitalist economic system requiring competition and revenue for survival. This means that, despite good intentions, healthcare providers can also be influenced to work or not work with a company based on how well it supports clinical revenue or brings in new patients, rather than patient care itself (DuBois, 2017; Spurling et al., 2010). We must acknowledge that money plays a role in these partnerships; the reality is that companies need to be financially sustainable to continue their operations.

Thus, physicians are tasked with discerning conscious and unconscious sources of reasonable bias (e.g., excellent support in patient care) from potentially harmful sources (e.g., flashy sales tactics). Further, this study observed that while some physicians recognized bias as a significant concern, others did not acknowledge its impact, suggesting that some physicians may lack the skills to identify and respond to biases effectively (Robbins, 2018; Robbins et al., 2019). It is important to address the fact that biases are ubiquitous to humanity, regardless of personality, intelligence, or professional experience (Saposnik et al., 2016). To responsibly operate within this context, companies and physicians must work together to create a system that benefits patients and the public while remaining financially sustainable. Hospitals can play a key role when they provide their physicians with training on recognizing and evaluating sources of bias from industry and systems to counteract them (Breault, 2015; Sah and Fugh-Berman, 2013). Additionally, the opportunity for industry influence on patient care decisions may decrease as new competitors enter the neurotechnology market if physicians and patients have greater access to more options among devices. Future research should monitor this trend, including whether industry ends up needing to provide more data about a device to convince physicians and patients that they have a preferable product.

Physicians reported several evidence-based and policy-compliant strategies that they regularly employ to support objective decision-making. The strategies offered suggest that educational efforts and policy enforcement may have a positive impact on physician behavior. However, this conclusion requires additional research to substantiate. Little is currently known about the impact that conflict of interest training and policy has on physician beliefs and behavior concerning bias; much of the literature in this area is outdated, more recent studies report little evidence of physician behavior change, and there is a dearth of studies examining neurotechnology physician-industry relationships specifically (Lexchin and Fugh-Berman, 2021; McIntosh and DuBois, 2020; US Institute of Medicine Committee on Conflict of Interest in Medical Research Education Practice, 2009). Further, physician-industry collaboration is essential for neurotechnology devices to be purchased, programmed, implanted, or maintained, often to an extent that exceeds the required involvement of most pharmaceutical interventions (Chatterji et al., 2008; Illes et al., 2020).

Some physicians in this study reported self-monitoring and limiting their interaction with industry to promote objective decision making. While this may appear to be a helpful strategy, good intentions and self-monitoring alone are unlikely to be sufficient to avoid bias. Avoiding industry interaction altogether is not possible for the clinical use of many neurotechnologies, and doing so means that patients in need may never access beneficial treatments. Methods to limit, but not prohibit, interactions between physicians and neurotechnology companies, is an area that would benefit from further investigation. A deeper examination of what specific strategies are effective in reducing bias is also pressing area for future research to establish concrete strategies for striking a balance that allows physician-industry relationships while mitigating risk of undue influence on clinical decisions.

### 4.1 Limitations

Several limitations must be considered when interpreting interview findings from this study. While the sample size (*N* = 13) is sufficient to qualitatively characterize physician experiences and perspectives in their relationships with neurotechnology companies, this sample size precludes statistical comparisons and thus constrains the generalizability of the results (Guest et al., 2006). Additionally, the findings of this study report the perceptions of physicians regarding relationships with the neurotechnology industry and do not reflect directly measured effects on clinical or patient outcomes. All physicians in this study had ties to industry in some capacity, and most (92%) were also engaged in research involving neurotechnology industry partners. Therefore, the results of this study may not sufficiently represent the perceptions of physicians who solely provide clinical care to patients and who do not have experience working with neurotechnology companies. Further, the counts reported for themes throughout this article provide the prevalence of ideas observed during interviews, but higher code counts do not necessarily equate to relative importance.

## 5 Conclusion

Given the rapid expansion of neurotechnologies in clinical care, this study reveals vital insights into physician relationships with neurotechnology companies and carries implications for policies, practices, and organizational operations. Both healthcare providers and device companies operate within a capitalist system where financial motivations may intersect with patient care priorities, requiring efforts on behalf of both parties to facilitate responsible and productive relationships. Neurotechnology companies can foster reliable clinical relationships through robust customer support systems that leverage prompt and transparent communication. Companies’ ongoing self-assessment and adaptation to patient and physician needs can lead to better practices, long-term sustainability, and stronger physician partnerships. Physician training and systematic approaches are necessary to help mitigate biases that impact clinical decision-making. Further research is needed to understand and develop strategies that allow beneficial physician-industry interactions without compromising objectivity. By working together, physicians and neurotechnology companies can create systems that prioritize patient well-being while maintaining financial viability, ensuring the continued progress and accessibility of neurotechnological advancements.

## Data Availability

The datasets presented in this study can be found in online repositories. The names of the repository/repositories and accession number(s) can be found below: Qualitative Data Repository: https://doi.org/10.5064/F6J4JOSG.
